# Impact of Pharmacogenomics in Clinical Practice

**DOI:** 10.3390/ph16111596

**Published:** 2023-11-13

**Authors:** Nicola Principi, Kyriakoula Petropulacos, Susanna Esposito

**Affiliations:** 1 University of Milano, 20122 Milan, Italy; nicola.principi@unimi.it; 2 Amici del Bambino Malato ODV, 41121 Modena, Italy; ptrkrk63m68f257v@gmail.com; 3 Pediatric Clinic, Department of Medicine and Surgery, University Hospital of Parma, 43126 Parma, Italy

**Keywords:** drug prescription, drug-related adverse events, genetic variants, pharmacogenomics, pharmacokinetics

## Abstract

Polymorphisms of genes encoding drug metabolizing enzymes and transporters can significantly modify pharmacokinetics, and this can be associated with significant differences in drug efficacy, safety, and tolerability. Moreover, genetic variants of some components of the immune system can explain clinically relevant drug-related adverse events. However, the implementation of drug dose individualization based on pharmacogenomics remains scarce. In this narrative review, the impact of genetic variations on the disposition, safety, and tolerability of the most commonly prescribed drugs is reported. Moreover, reasons for poor implementation of pharmacogenomics in everyday clinical settings are discussed. The literature analysis showed that knowledge of how genetic variations can modify the effectiveness, safety, and tolerability of a drug can lead to the adjustment of usually recommended drug dosages, improve effectiveness, and reduce drug-related adverse events. Despite some efforts to introduce pharmacogenomics in clinical practice, presently very few centers routinely use genetic tests as a guide for drug prescription. The education of health care professionals seems critical to keep pace with the rapidly evolving field of pharmacogenomics. Moreover, multimodal algorithms that incorporate both clinical and genetic factors in drug prescribing could significantly help in this regard. Obviously, further studies which definitively establish which genetic variations play a role in conditioning drug effectiveness and safety are needed. Many problems must be solved, but the advantages for human health fully justify all the efforts.

## 1. Introduction

For many years, it has been established that in a great number of patients the expected efficacy, tolerability, and safety of most medicines could be achieved only when drug prescribing could be individualized. It is well known that several factors, such as age, sex, disease characteristics, environmental factors, and diet, can significantly influence drug pharmacokinetics and pharmacodynamics and that, when the relevance of one or more of these factors in each patient is not carefully considered and traditionally recommended dosages are not individually adjusted, the impact of drug therapy can be different from that which is desired [1]. However, clinical effectiveness can be lower and the risk of severe drug-related adverse events significantly higher than expected. Traditionally reported examples in this regard are the need to adjust the dosage of some drugs in patients with reduced renal function [2], in those with severe liver disease [3], and in neonates and younger infants [4,5]. More recently, the importance of personalized drug dosage has been further highlighted by the evidence that the impact of drug administration could be strictly dependent on genetic factors and that genetic variants could contribute up to 95% to determining the interindividual variability in drug responses [6].

Several studies have shown that polymorphisms of genes encoding drug metabolizing enzymes and transporters can significantly modify the absorption, distribution, metabolism, and elimination of medicines, and this can be associated with significant differences in drug efficacy, safety, and tolerability [7]. Moreover, genetic variants of some components of the immune system, mainly human leukocyte antigens (HLAs) and T-cell receptors (TCRs), can explain clinically relevant drug-related adverse events [8]. All these findings have strongly increased the interest in pharmacogenomics, and several drug regulatory agencies, including the European Medicines Agency (EMA) [9] and the U.S. Food and Drug Administration (FDA) [10], consider studies of genetic factors that cause variability in drug response an essential part of the process of developing and authorizing drugs. Furthermore, it has definitively established that correlations between genetic variants and clinical effects should be systematically included in the package leaflet of all the drugs for which this information is known. In the USA, it occurs in more than 100 commercially available drugs [11]. Finally, to translate pharmacogenomics into clinical practice, several pharmacogenomics consortia, including the Clinical Pharmacogenetics Implementation Consortium (CPIC), have been created [12,13,14,15]. These institutions publish genotype-based drug guidelines to help clinicians understand how available genetic test results could be used to optimize drug therapy in each patient, according to the characteristics and frequency of genetic polymorphisms in the treated population. With this information, a few hospitals have included pharmacogenomic tests in routine clinical practice to promote genetic-guided precision medicine at least in some selected patients [16,17]. However, the implementation of drug dose individualization based on pharmacogenomics remains scarce, although it has been evidenced that about 10% of children receive one drug for which a change in prescribing due to genetic variants could be recommended [18]. In this narrative review, the impact of genetic variations on the disposition, safety, and tolerability of the most commonly prescribed drugs is reported. Moreover, reasons for the poor implementation of pharmacogenomics in everyday clinical settings are discussed.

## 2. Genetic Variations and Impact on Drug Transportation and Metabolism

### 2.1. Normal Mechanisms of Drug Transportation and Metabolism

The activity of transporters and that of metabolizing enzymes play an essential role in conditioning the pharmacokinetics of most drugs. Transporters are proteins that regulate the movement of drugs into and out of the various tissues and fluid compartments, maintaining homeostasis and controlling drug access to metabolizing enzymes and excretory pathways [19]. Among transporters, the most important are the Solute Carrier Superfamily (SLC) and the ATP-Binding Cassette superfamily (ABC) [20]. Among these, those with common polymorphisms are SLC22A1, ABCB1, ABCC2, ABCG2, and SLCO1B1, SLCO1B3, ABCB1, and ABCC2 [21].

To be detoxified and more easily excreted, most drugs undergo chemical modifications that occur in various organs and body systems, mainly the liver, through the activity of several enzymes [22,23]. These metabolic processes are categorized as phase I and phase II drug metabolism. Phase 1 reactions convert a parent drug to more water-soluble active metabolites by unmasking or inserting a polar functional group (−OH, −SH, −NH2). Among the phase 1 metabolizing enzymes, those included in the cytochrome P (CYP) 450 family, particularly CYP2C9, CYP2C19, and CYP2D6, are the most important. They are responsible for the metabolism of about 80% of commonly prescribed drugs [22], and variations in these genes influence the metabolism of 60% of these drugs [23]. Phase 2 reactions result in the conjugation of the drug with an endogenous substance by acetylation, glucuronidation, sulfation, and methylation with the formation of an inactive metabolite. Uridine diphosphate glucuronosyltransferase (UGT), glutathione S-transferase (GST), sulfotransferase, N-acetyltransferase (NAT), and thiopurine methyltransferase (TPMT) are the most common phase 2 metabolizing enzymes and those with more frequent genetic polymorphisms.

According to the impact of polymorphisms on gene expression, patients can have reduced or increased drug metabolization and are classified as poor metabolizers (PMs), intermediate metabolizers (IMs), extensive (EMs) metabolizers, and ultrarapid metabolizers (UMs) [24,25]. As most metabolizing processes lead to drug inactivation, RMs and UMs receiving recommended drug doses have generally reduced drug effects, including the reduced risk of adverse events. The opposite occurs when an inactive drug is given, and metabolization is associated with the synthesis of an effective, potentially toxic, molecule.

### 2.2. Impact of Genetic Variants on Pharmacokinetics

A great number of drugs can have different transportation and metabolism due to genetic variants. The list of these pharmacogenetic associations can be found on the websites of several institutions, including the U.S. Food and Drug Administration website [10]. The most important examples in this regard, with particular attention to those leading to variations in drug use, are reported below. Table 1 summarizes the enzymes involved in phase I and phase II drug metabolism.

#### 2.2.1. Polymorphisms of the Most Important Phase I Metabolism Enzymes

CYP2C9 metabolizes approximately 25% of clinically administered drugs. The prevalence of PMs ranges from 3 to 4% in Southern Europe and the Eastern Mediterranean coast to <1% in Asian and African populations, except for Emiratis (11.1%) [26]. Among the drugs metabolized by CYP2C9, there are the anticoagulant S-warfarin, the anticonvulsant phenytoin, some nonsteroidal anti-inflammatory drugs (NSAIDs), and some hypoglycemic agents such as glipizide and tolbutamide. In some cases, poor metabolization leads to severe clinical problems. When usual doses of warfarin are used, in PMs, the risk of internal bleeding is greatly increased [27]. To reduce this problem, a number of dosing algorithms incorporating point-of-care genotyping information leading in most cases to an improved anticoagulation control were developed [28,29]. One of these was tested, with favorable results in children [30]. Similarly, dose adjustments are needed in adult patients receiving phenytoin [31]. Generally, it is recommended that PMs are given a traditional first dose but that, for subsequent doses, typical maintenance doses are reduced by about 50% and continuously adjusted according to therapeutic drug monitoring, response, and side effects [32].

Regarding NSAIDs that are metabolized by CYP2C9 (i.e., ibuprofen, celecoxib meloxicam, flurbiprofen, piroxicam), data indicate that in PMs, the drug’s half-life is significantly increased, with an increase in the risk of drug-related adverse events. In these subjects, it is recommended to initiate treatment at 25–50% of the traditional dose or use NSAIDs not metabolized by CYP2C9 (i.e., acetylsalicylic acid, ketorolac, naproxen, sulindac) [33]. No further variation is required during pregnancy because the clearance of drugs metabolized by CYP2C9 does not vary during pregnancy [34]. An exception might be extremely preterm infants. A study has shown that the administration of ibuprofen to treat patent ductus arteriosus was not followed, contrarily to what was expected, by an increased drug efficacy, supporting the hypothesis that in extremely preterm babies, the clinical response to ibuprofen is not related to CYP2C9 genotype [35]. Table 2 summarizes the main drugs whose metabolism can significantly change due to CYP2C9 gene variations.

The prevalence of CYP2C19 PM metabolism phenotype is 2–5% among Caucasians and Africans and ~15% in Asians. On the contrary, URs can be identified in 18–28% of European populations, in 17–18% of African populations, and in 0.3–4% of Asian populations [36]. Diazepam, proton pump inhibitors, voriconazole, and clopidrogel are included among drugs whose levels are influenced by CYP2C19 genetic polymorphisms. Systemic drug exposure to diazepam can vary by more than sixfold between individuals. Standard doses may be poorly effective. In PMs, on the contrary, recommended diazepam doses can lead to higher-than-expected drug levels with extensive sedative effects [37]. However, at the moment, this adverse event is not reported on the drug’s label unless the drug is given with other medicines such as cimetidine, ketoconazole, fluvoxamine, fluoxetine, and omeprazole that inhibit CYP2C19 expression [38]. The metabolization of omeprazole to 5-hydroxy omeprazole and omeprazole sulphone can vary significantly. In Ems, increased metabolization rapidly reduces drug concentrations and leads to poor clinical response, as evidenced in patients with *Helicobacter pylori* infection [39,40]. The antiplatelet activity of clopidogrel is significant influenced by CYP2C19 activity, as the enzyme converts the prodrug into an active drug. In PMs, the increased threat of frequent stroke, stent thrombosis, and myocardial infarction have been reported [41,42]. Regarding voriconazole, a synthetic triazole which is included among the first-line antifungal drugs, it has been reported that PMs, including children, receiving standard doses are at an increased risk of adverse events (hepatotoxicity, visual hallucinations, and encephalopathy) [43]. For this, in these individuals, the choice of an alternative agent independent from CYP2C19 or the use of a lower dose associated with careful therapeutic drug monitoring are recommended. Table 3 describes the main drugs whose metabolism can significantly change due to CYP2C19 gene variations.

CYP2D6 actively metabolizes approximately 20–25% of all administered drugs [44], including drugs for pain management, cancer, mental health disorders, antiarrhythmics, and β-blockers [45]. The prevalence of CYP2D6 gene polymorphisms varies significantly between populations. PMs have been identified in 0.4–6.5% of individuals, with the highest values in European and American Caucasians and the lowest in East Asian, Oceanian, and Middle Eastern populations. The UM phenotype occurs in 1–2% of patients, although studies have reported that it is present in up to 28% of North Africans, Ethiopians, and Arabs; up to 10% in Caucasians; 3% in African Americans; and no more than 1% in Hispanic, Chinese, and Japanese populations [46].

A good example of the clinical impact of CYP2D6 gene polymorphisms is given by the studies regarding psychiatric drugs [47]. It has been shown that in subjects with gene mutations, risperidone and aripiprazole metabolism was significantly changed. In PMs and IMs, exposure to active drugs after recommended doses was increased, and a substantial reduction in dosage was required to maintain normal blood levels. On the contrary, in UMs, drug levels were inadequate to obtain favorable clinical results [48]. However, the most clinically relevant example of the impact of CYP2D6 genetic variations on drug metabolism in children is given by codeine. This opioid is converted by CYP2D6 into its active metabolite, morphine, which is truly responsible for the clinical efficacy, safety, and tolerability of the drug. PMs convert only 10% of codeine to morphine, whereas this occurs in 40% and 51% of EMs and UMs, respectively. Consequently, pain relief is generally very poor in PMs that have two inactive copies of CYP2D6 and produce low morphine concentrations. The prescription of an alternative analgesic is recommended for these subjects. On the contrary, in EMs and particularly in UMs, which convert codeine to morphine more rapidly and more completely, the control of pain is very good but the high morphine levels can lead to severe adverse events such as extreme sleepiness, confusion, and shallow breathing, in some cases so severe that they can be fatal [49]. This explains why codeine use is contraindicated in children under 12 years of age in most countries [50,51]. Fortunately, as CYP2D6 gene expression matures as early as 2 weeks after birth, the effect of genetic variations does not change during child development [52].

#### 2.2.2. Polymorphisms of the Most Important Phase II Metabolism Enzymes

Several studies have shown that conjugation with glucuronic acid trough UGT enzyme activity is essential not only for the clearance and detoxification of several endogenous compounds (bilirubin, steroids, thyroid hormones, neurotransmitters, fatty acids) but is relevant also for the metabolization of a great number of commonly used drugs, such as paracetamol, some nonsteroidal anti-inflammatory drugs (naproxen, flurbiprofen, indomethacin, diclofenac), several neurologic drugs (anticonvulsants, antipsychotics, and benzodiazepine), and some anticancer drugs [53]. Old studies carried out on subjects with UGT gene polymorphisms have shown that these gene variations, despite being very common [54] and the cause of clinical syndromes with high unconjugated hyperbilirubinemia levels (i.e., Gilbert’s disease and Crigler–Najjar syndrome) [55], do not play a relevant role as a cause of drug clearance modification. Mutations were not associated with significant alterations in valproate [56], zidovudine, morphine, or codeine metabolism [57]. A substantial reduction in benzodiazepine clearance initially reported in individuals carrying the UGT2B15*2 variation [58] was not confirmed. However, the results of recent studies seem to suggest that the metabolism of some anticancer drugs is significantly affected by some UGT polymorphisms. In PMs, the administration of irinotecan has been found to be associated with higher systemic active metabolite concentrations with a higher risk of severe adverse events, such as profuse diarrhea and severe or life-threatening neutropenia [59]. Similar problems were found in PMs with the UGT1A1 *28/*28 genotype receiving sacituzumab govitecan-hziy [60]. Moreover, hyperbilirubinemia has been reported in patients with UGT polymorphisms receiving nilotinib [61] or pazopanib [62]. Finally, and this is the most relevant example of the risk of severe clinical problems in patients with polymorphisms of genes encoding for enzymes of the phase II metabolism pathway, it has been repeatedly evidenced that thiopurine S-methyltransferase (TPMT) genetic variants can be associated with significant modifications in thiopurine metabolism, leading to a reduced tolerance of these drugs. Thiopurine drugs, azathioprine, 6-mercaptopurine, and 6-thioguanine, are widely used to treat cancer, onco-hematological diseases, and autoimmune diseases and to prevent transplant rejection [63]. TPMT, together with nudix hydrolase, is the most important enzyme for thiopurine metabolism [64], and TPMT activity is inversely correlated with the levels of the active metabolites, as observed in children with leukemia [65] and in patients with inflammatory bowel diseases [66]. Patients with high TPMT activity are at risk of poor treatment efficacy due to a low production of active metabolites. The opposite occurs in PMs, who are at an increased risk of adverse events such as myelosuppression, gastrointestinal intolerance, pancreatitis, and hypersensitivity [67]. As up to 14% of the population is known to have a decreased TPMT activity and 0.3% has no TPMT activity, the determination of the patient’s TPMT genotype or phenotype before thiopurine administration is strongly recommended in order to decide which patients should not be given these drugs and which should receive reduced drug doses to maintain therapeutic effects [68].

#### 2.2.3. Polymorphisms of the Most Important Transporters

A limited, if any, role of transporter gene variations on drug disposition has been shown. Data regarding ABCB1 variants are inconsistent. Moreover, although the polymorphism of ABCG2 has been associated with modifications in statin bioavailability, and ABCC2 variants have been shown to be the cause of reduced methotrexate and statin disposition [69], none of these biomarkers are currently used for drug dosage optimization. Significant evidence that polymorphisms of ABC efflux transporters can have severe clinical consequences is lacking [70]. Similar conclusions can be drawn regarding SLC gene polymorphisms. Studies regarding their impact on drug disposition are conflicting, as evidenced by the results of studies concerning metformin. Whereas Raj et al. did not find any effect of the SLC47A1 and SLC47A2 gene polymorphisms on the glycemic response to metformin [71], Chen et al. reported that in patients with some SLC47A1 and SLC47A2 variations, metformin administration was poorly effective [72]. Not even the demonstration that allowed us to consider this type of genetic variation was sufficient to modify the usual indications for use. Further studies are needed to exactly quantify the real role of transporter genetic variations in drug disposition.

## 3. Genetic Variants That Affect Immune Response to Drugs

Immune-mediated adverse drug reactions account for about 20% of all adverse drug reactions. Most of them depend on HLA polymorphism [73]. Polymorphic HLA produces >10,000 HLA class I genetic variants and >4500 HLA class II chain genetic variations. Variants may modify specific immune responses with the development of abnormal reactions, such as autoimmune diseases [73]. Practically, drugs interact with certain HLA variants forming an immunogenic complex that is recognized by the immune system and evokes an immune reaction, leading to the development of drug-related adverse events. As the number of possible HLA–drug combinations is very high, HLA-mediated adverse events can only rarely be predicted. Prediction is further complicated by the evidence that, in the same subject, more than one HLA polymorphism influencing the safety of a single drug can be present and that, in some cases, these polymorphisms can be protective. An example of multiple HLA alleles influencing the risk for certain adverse drug reactions is allopurinol, for which the HLA-B*58:01 allele is the most common cause of severe cutaneous reaction, but the HLA-Cw*03:02 allele can also play a role [74]. Protective HLA polymorphisms have been reported for several drugs, such as beta-lactam antibiotics, cotrimoxazole, acetaminophen, and clozapine. For carbamazepine, in the same individual, well-known alleles (HLA-B*15:02, HLA-A*31:01) associated with severe skin reactions, such as Stevens–Johnson syndrome (SJS) and toxic epidermal necrolysis (TEN), and HLA alleles (HLA-B*40:01, HLA-Cw*01:02, and HLADRB1*04:05 for SJS/TEN, HLA-B*15:01 for SJS/TEN, HLA-B*40:01 for SJS/TEN and drug reaction with eosinophilia and systemic symptoms (DRESS), HLA-B*46:01 for all the severe acute skin reactions) that are reported to confirm a certain degree of protection can be present at the same time [75].

Most HLA polymorphism-mediated adverse events involve the liver and the skin [76]. Fortunately, in most of the cases, they have poor clinical relevance, and manifestations are generally resolved in a few days after drug therapy has been suspended. However, repeated administration can lead to more severe disease, suggesting that a careful medication history may reveal important information regarding the safety of a given drug. However, in some cases, particularly those that are very severe, immune-mediated drug-related diseases can occur without any previous history. This is the case of acute liver failure [77] and the most severe drug reactions, such as SJS, TEN, DRESS, and maculopapular exanthema (MPE) [78]. A great number of drugs have been associated with immune-mediated adverse events [79,80]. Those for which the risk of severe adverse events is relatively common and is generally reported in the package leaflet of the drug, although with differences between countries, are listed in Table 4 [80,81,82,83,84,85,86]. The main genetic variations associated with the abnormal immune response and the associated phenotype are also listed.

**Figure 1:**
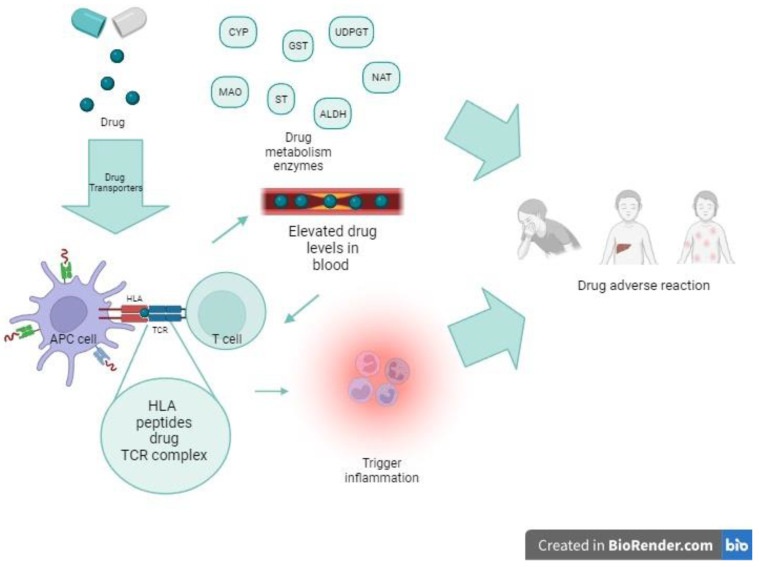
How genetic variants affect immune response to drugs (created with BioRender.com; accessed on 21 October 2023).

In addition to HLA alleles, specific TCRs have been associated with adverse event development (Figure 1).

Examples in this regard have been collected in patients with carbamazepine-induced SJS/TEN and can explain why the frequency of SJS/TEN can be quite similar between some populations despite the frequency of HLA polymorphism associated with these diseases being different [87]. Interactions involving an HLA–drug–TCR are considered essential for inducing some type B idiosyncratic adverse events. This further complicates the prevision of the drug-related problems in a single patient.

## 4. Implementation of Pharmacogenomics

Knowledge of the genetic characteristics of a patient allows us to define whether an indicated drug can be efficacious, whether the patient is at an increased risk of developing severe drug-related adverse events, and finally, what the optimal drug dosage is. To help clinicians understand how available genetic test results should be used to optimize drug use, several guidelines concerning drugs whose disposition and safety are influenced by pharmacogenomics have been prepared [88]. Moreover, several methods for developing and applying pharmacogenomics and personalizing drug therapy have been proposed [89]. Despite this, the implementation of pharmacogenomics in routine clinical practice has been sparse, and very few centers currently include pharmacogenetic tests in routine clinical care [90,91,92,93,94,95]. Several factors can explain this finding. A role can be played by the lack of precise information on the real frequency of genetic variations involved in drug disposition or adverse event determination in different populations, particularly those with less advanced health systems. The lack of a shared definition of the level of evidence that is necessary to implement pharmacogenetics-based information into clinical care also seems to be important. Organizations that curate pharmacogenetic evidence, including the CPIC and FDA, differ significantly in their interpretation of the available pharmacogenetic data, and this explains, at least in part, why pharmacogenomics recommendations to personalize therapy from medical societies are different and controversial [96]. Some experts think that, for each drug, before implementation, the results of randomized controlled trials (RCTs) clearly showing that personalized therapy guarantees superior benefits than the standard therapy should be collected [97,98]. The results of RCTs are actually available for several drugs (acenocoumarol, phenprocoumon, clopidogrel, statins, warfarin, proton pump inhibitors, azathioprine, tacrolimus, thiopurines, abacavir, highly active antiretroviral therapy (HAART) regimens, isoniazid, nonsteroidal anti-inflammatory drugs and opioids, multiple anti-depressants, multiple neuropsychiatric medications, nortriptyline, and venlafaxine), and in most of them, relevant benefits of pharmacogenomics-guided therapeutic strategies have been shown. Data collected through the European Ubiquitous Pharmacogenomics Clinical Implementation Project indicate that, applying the recommendations of the Royal Dutch Association for the Advancement of Pharmacy—Pharmacogenetics Working Group, patients with pharmacogenomics-actionable test results experience less severe drug-related adverse events (21.0%) than controls receiving standard treatment (27.7%) [99]. Moreover, the use of pharmacogenomics testing was found to be associated with reduced hospitalization rates for drug toxicity, improved drug efficacy, and reduced costs of medical assistance [100,101]. Unfortunately, the available data collected with RCTs are frequently debated due to the methodological limitations of most of the studies. The most important limit is that the number of patients enrolled is too low to allow reliable statistical analyses. Because only a part of the enrolled subjects carries the gene variation(s) related to the drug’s reduced effectiveness or increased toxicity, a number of patients greater than that needed for a typical drug RCT is required when pharmacogenomics is evaluated. This does not occur in most of the available RCTs [102]. Moreover, RCT results can be conflicting and make it difficult to decide whether specific pharmacogenetic testing must be recommended in patients receiving a given drug. Typical in this regard is the case of CYP2D6 testing in patients receiving tamoxifen. This test was initially recommended by some experts due to the supposed impact of the CYP2D6 variant on drug efficacy. However, the test is presently not routinely offered by oncologists prior to prescribing tamoxifen due to the evidence of severe genotyping errors in several studies, making the results unreliable [103]. On the other hand, even the data on the possible economic advantages of the use of pharmacogenomics are not always indicative of the importance of implementation programs. The analysis of 108 studies evaluating 39 drugs revealed that pharmacogenomics was cost-effective in 48 (44.4%) and cost-saving in 29 (26.8%). The smallest benefits were found in studies evaluating HLA testing for abacavir, allopurinol, or carbamazepine/phenytoin. A total of 26 studies were performed, but the cost-efficacy or cost-saving of the test was shown only in 15 (57.7%) [104].

Some limits of RCTs could be overcome by performing a very large initial screening in order to evaluate the importance of pharmacogenomic testing only in patients with a known genetic variation. But this method is also debatable as it raises important ethical limitations. If the variant under study is associated with an increased risk of life-threatening adverse events, as in the case of carbamazepine-induced severe cutaneous reactions in patients with an HLA*15:02 allele, the inclusion of patients at risk in the control group receiving standard therapy is deemed to be unethical [105,106]. In any case, whatever the method used, there is no doubt that verifying the benefits of the introduction of pharmacogenomics in clinical practice can be very expensive and discourage research, especially when it concerns rarely used drugs and relatively uncommon genetic variants. Moreover, the implementation of pharmacogenomics testing in a center remains very difficult as it entails several steps and involves several stakeholders. The choice of the drug–gene pairs to monitor should be made considering which tests are authorized or required by national drug regulators and the type of patients that are commonly followed in the center. This choice is made by the local pharmacy and therapeutics committees, and the hospital laboratory and information technology department should be involved in conducting possible gene and drug analyses. Finally, healthcare providers should decide which patient should be tested, analyze the test results, make the appropriate therapeutic decisions, and explain them to the patient. The complexity of this organization, the high management costs, and the obligation to explain to patients the usefulness of unusual tests make the implementation of pharmacogenomics testing even more difficult [107].

Another consideration regarding the poor implementation of pharmacogenomics in clinical practice regards the poor knowledge about this method for medicine personalization by health care providers. A recent survey of the inclusion of pharmacogenomics in medical and pharmacy study programs showed that in only about 10% of cases pharmacogenomics was considered a mandatory subject [108].

**Figure 2:**
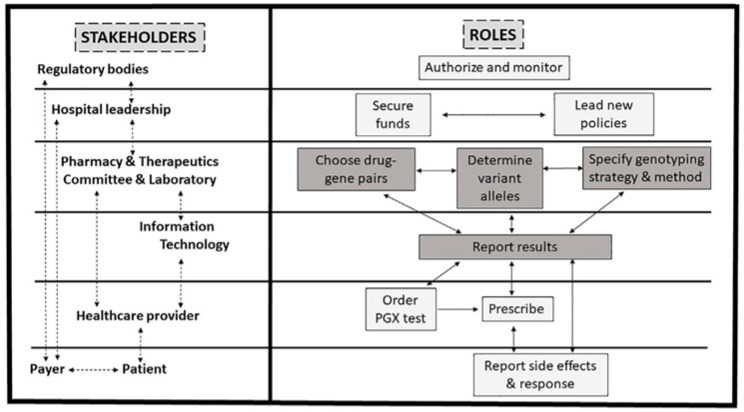
Proposed framework for pharmacogenomics (PGx) implementation in clinical practice.

The implementation of drug dose individualization programs and the prediction of effective and safe drug dosages is further complicated in pediatrics by the relative expression of some genes in the early developmental stages. The differentiation of drug metabolism between subjects with genetic variants conditioning poor or no metabolic function and those with the wild-type genotype can be very difficult or totally impossible when gene activity is poorly expressed. Only later, when enzyme activity is completely matured, the effect of polymorphism can be identified. Data collected in term and preterm infants receiving pantoprazole are a good example of the impact of ontogeny on genotype–phenotype discordance. Pantoprazole, used to treat gastroesophageal reflux, is a substrate for the CYP2C19 enzyme. In adult PMs, the systemic exposure to pantoprazole increases up to fivefold in the presence of a nonfunctional enzyme, as in this case drug clearance is reduced [109]. On the contrary, in neonates, drug clearance did not substantially differ when data collected in subjects with a CYP2C19 genotype considered predictive of an extensive metabolization were compared to those without [110]. Genotype–phenotype concordance may become apparent by ~15 weeks postnatal age. Similar findings were reported when CYP2C9 activity was studied. The activity of this enzyme was found to be very low in the fetus during the first trimester (1–2%) and at term (30%) and reached adult values only between five months and two years of age [111]. From this, the recommendation was given to use lower doses of phenytoin in neonates and younger infants (5 mg/kg/day), increasing the dosage (8–10 mg/kg/day) only after the 5th month of age, proportionally to the increase in enzyme activity [4]. Figure 2 shows a possible framework for PGx implementation in clinical practice.

## 5. Conclusions

Several examples indicate that personalized medicine can significantly improve therapy, disease prevention, and health maintenance in a great number of individuals. Knowledge of how genetic variations can modify the effectiveness, safety, and tolerability of drugs can lead to an adjustment in usually recommended drug dosages, an improvement in effectiveness, and a reduction in drug-related adverse events. Despite some efforts to introduce pharmacogenomics in clinical practice, presently very few centers routinely use genetic tests as a guide for drug prescription. This is because several factors, among which the most important seem to be the poor knowledge of the frequency of genetic variations in a given population, the clinical impact of the use of pharmacogenomics, the complexity of the pharmacogenomics implementation, and the relevance of costs, may discourage local health authorities from personalizing medicine using genetic information. The education of health care professionals seems to be critical to keep pace with the rapidly evolving field of pharmacogenomics. The gap between geneticists and clinicians should be reduced. Clinicians should understand that pharmacogenomics is only one of the variables that should be considered when personalizing drug prescriptions. Clinicians usually take into account age and body system functions when they prescribe drugs and must also learn to use genetic information for this purpose. Multimodal algorithms incorporating both clinical and genetic factors could significantly help in this regard. Obviously, further studies definitively establishing which genetic variations play a role in conditioning drug effectiveness and safety are needed. Moreover, further research should focus on the implementation of pre-emptive pharmacogenetic panel testing for patients with chronic disease requiring long-term treatment with one or more drugs for which an actionable drug–gene interaction has been reported and on the assessment of the true incidence of the studied genetic variances in different populations. Furthermore, since the majority of pharmacogenetics recommendations are based on the estimation of single drug–gene interactions, for older people with polypharmacy, it is imperative that methods and tools for the prediction of multiple drug–drug–gene interactions are developed. Several tests, potentially for direct oral anticoagulants, beta-blockers, or antihypertensives, should be systematically planned. Finally, studies are needed on gene therapy in order to identify subjects with polymorphisms of the genes involved in drug metabolism. Many problems need to be solved, but the advantages for human health fully justify all the efforts.

## Figures and Tables

**Table 1:** Enzymes involved in phase I and phase II drug metabolism.

Phase 1 Enzymes	Phase 2 Enzymes
Cytochrome P450 Monooxygenase (CYP)	Uridine diphosphate glucuronosyl Transferase (UDPGT)
Flavin-containing Monooxygenase	Sulfo transferase (ST)
Esterase	N-Acetyl transferase (NAT
Alcohol Dehydrogenase (ADH)	Glutathione S-Transferase (GST)
Aldehyde Dehydrogenase (ALDH)	Methyl Transferase
Monoamine Oxidase (MAO)	Amino Acid Conjugation

**Table 2:** Main drugs whose metabolism can significantly change due to CYP2C9 gene variations.

Drug	Therapeutic Area	Clinical Impact
S-warfarin	Cardiovascular diseases	When usual doses of warfarin are used, in PMs, the risk of internal bleeding is greatly increased. Drug dose should be established according to CYP2C9 polymorphism genotype. IMs should use 65% of the standard initial dose; PMs 20%. Drug monitoring is recommended to establish maintaining doses.
Phenytoin	Neurology	PMs are at greater risk of developing CNS adverse effects as well as serious cutaneous adverse reactions when given usual dosages of phenytoin. It is recommended to start with recommended doses and reduce maintaining doses by about 50%.
Some NSAIDs (ibuprofen, celecoxib, meloxicam, piroxicam, flurbiprofen, mefenamic acid)	Diseases with inflammation	In PMs, increased risk of gastrointestinal ulcers, serious cardiovascular events, hypertension, acute renal failure, and worsening of preexisting heart failure. In these patients, it is recommended to initiate treatment at 25–50% of the traditional dose or use NSAIDs not metabolized by CYP2C9 (acetylsalicylic acid, ketorolac, naproxen, sulindac).
Some hypoglycemic drugs (glipizide, tolbutamide)	Diabetology	These drugs are a substrate of the genetically polymorphic enzyme CYP2C9. However, the pronounced differences in pharmacokinetics due to the variants did not significantly affect plasma insulin and glucose concentrations. No dose variations are needed.

**Table 3:** Main drugs whose metabolism can significantly change due to CYP2C19 gene variations.

Drug	Therapeutic Area	Clinical Impact
Diazepam	Neurology and psychiatry	In PMs, standard doses can lead to increased risk of sedation and unconsciousness. Plasma half-life of the drug is about up to six times longer than in individuals homozygous for wild-type CYP2C19 genotype. However, modification of dosage is not required unless drugs that inhibit CYP2C19 gene expression are given at the same time.
Proton pump inhibitors	Gastroenterology	Increased and decreased drug effectiveness in PMs and EMs, respectively.
Clopidrogel	Cardiology	In PMs, drug activity is reduced, leading to increased risk of cardiovascular events.
Voriconazole	Infectious diseases	In PMs, standard doses can lead to increased incidence of severe adverse events. In these patients, alternative drugs or use of lower doses with careful monitoring of plasma levels are recommended.

**Table 4:** Main drugs associated with severe immune-mediated adverse events.

Drug	Genetic Marker	Associated Manifestations
Abacavir [80]	HLA-B*57:01	Development within 6 months from starting therapy. Symptoms are fever, rash, nausea, vomiting, diarrhea or abdominal pain, and fatigue and malaise. Occasionally, respiratory symptoms are prominent and pneumonia occurs. Frequency of polymorphism is about 14% in Caucasian, 12.6% in Asian, 2.6% in South American, 2.2% in Mexican, and 1% in African populations. All patients should be screened for the genetic variation prior to initiating or reinitiating therapy with abacavir, unless patients have a previously documented HLA-B*57:01allele assessment.
Allopurinol [81]	HLA-B*58:01	DRESS, SJS/TEN. Common among Asian subpopulations, notably in individuals of Korean, Han-Chinese, or Thai descent. Presently, the FDA-approved drug label does not discuss HLA-B genotype. Testing for the HLA–B*58:01 allele prior to starting allopurinol is conditionally recommended for individuals of Southeast Asian descent (e.g., Han Chinese, Korean, Thai) and for African American individuals, over not testing for the HLA-B*58:01 allele. Universal testing for the HLA-B*58:01allele prior to starting allopurinol is conditionally recommended against in individuals of other ethnic or racial background over testing for the HLA-B*58:01allele.
Amoxicillin-clavulanate [83]	HLA-DRB1*-15.01	Drug-induced liver injury, mainly a transaminase increase.
Carbamazepine [84]	HLA-B*15:02 HLA-B*31:01	The clinical manifestations can vary widely, ranging from a mild skin rash, such as MPE and EEM minor, to severe diseases such as EEM major, SJS, TEN, DRESS, and AGEP. HLA-B*15.02 has been found mostly in Asian people but not in Caucasian patients. HLA-B*31:01is prevalent globally, particularly in indigenous populations of the Americas (Argentina 28.8%, Mexico 10.1%, the USA 7.8%, Nicaragua 6.7%, and Chile). Values of about 8% in Asia and varying from <1% to about 6% in Europe. FDA-approved labeling recommends HLA-B*15.02 screening before CBZ therapy in patients of Asian ancestry.

## Data Availability

Not applicable.
